# Cracking pattern of tissue slices induced by external extension provides useful diagnostic information

**DOI:** 10.1038/s41598-018-30662-9

**Published:** 2018-08-15

**Authors:** Keisuke Danno, Takuto Nakamura, Natsumi Okoso, Naohiko Nakamura, Kohta Iguchi, Yoshiaki Iwadate, Takahiro Kenmotsu, Masaya Ikegawa, Shinji Uemoto, Kenichi Yoshikawa

**Affiliations:** 1 Faculty of Life and Medical Sciences, Doshisha University, Kyotanabe, Kyoto Japan; 2 Department of Surgery, Graduate School of Medicine, Kyoto University, Kyoto, Japan; 3 Faculty of Science, Yamaguchi University, Yamaguchi Yamaguchi, Japan

## Abstract

Although biopsy is one of the most important methods for diagnosis in diseases, there is ambiguity based on the information obtained from the visual inspection of tissue slices. Here, we studied the effect of external extension on tissue slices from mouse liver with different stages of disease: Healthy normal state, Simple steatosis, Non-alcoholic steatohepatitis and Hepatocellular carcinoma. We found that the cracking pattern of a tissue slice caused by extension can provide useful information for distinguishing among the disease states. Interestingly, slices with Hepatocellular carcinoma showed a fine roughening on the cracking pattern with a characteristic length of the size of cells, which is much different than the cracking pattern for slices with non-cancerous steatosis, for which the cracks were relatively straight. The significant difference in the cracking pattern depending on the disease state is attributable to a difference in the strength of cell-cell adhesion, which would be very weak under carcinosis. As it is well known that the manner of cell-cell adhesion neatly concerns with the symptoms in many diseases, it may be promising to apply the proposed methodology to the diagnosis of other diseases.

## Introduction

Biopsy, or the inspection of tissue samples, is one of the most important methods for making a precise diagnosis in human disease, especially for evaluating the stage of malignant cancer. Various techniques for preparing tissue samples have been developed and pathologists describe the features of a specimen in terms of cell type, cellular arrangement, abnormality, morphology of the cell nucleus, etc.^1–10^. However, there is still some ambiguity in a diagnosis based on information obtained from the inspection of tissue slices. In addition, a great deal of skill is needed to give a precise pathological diagnosis. To realize quantitative pathological diagnosis, several attempts have been made to analyze images of pathological samples obtained by optical microscopy. These attempts have been based on pattern recognition or topology algorithms^11–15^, and also on information processing by so-called AI algorithms using “big data”^16^. In the present study, we tried to obtain additional information from sliced tissue sections for samples obtained from mice with disease in different pathological stages. We prepared sections of mouse liver. These mice were sacrificed to obtain whole liver samples at 36 weeks after 12 h of fasting from the last feeding under general anesthesia by inhalation of 1–2% isoflurane. Small pieces of the liver were cut from the whole liver and then snap-frozen in liquid nitrogen immediately. These sliced tissues were placed on an elastic sheet of polyurethane, instead of a glass slide, which is usually used in microscopic observation. Slices were then stretched under observation by optical microscopy. Interestingly, characteristic fine-cracking pattern was generated for the slice with Hepatocellular carcinoma, which is markedly different from those for the slices with non-cancerous steatosis. It has been shown that the analysis of cracking patterns can be used to derive index parameters^17–24^, which are quantitative parameters that are useful for obtaining a precise diagnosis.

## Materials and Methods

### Animal

Male 4 to 5 weeks old C57BL/6 mice were purchased from Japan SLC, Inc. (Shizuoka, Japan). All protocols were approved by the Ethics Committee of Animal Care and Experimentation at Kyoto University (MedKyo14250).

### Mouse NAFLD-HCC models

We examined liver tissues of mice that reflected different disease states: Healthy normal (Normal), Simple steatosis, Non-alcoholic steatohepatitis (NASH) and Hepatocellular carcinoma (HCC). Mice of the old C57BL/6 were induced to develop different disease states through a well-established procedure involving chemical additives in feed and other dietary interventions^25,26^. Mice were maintained at 24 ± 3 °C under a 12-h light/dark cycle with free access to water. Mice were fed a control diet or high fat diet (D12331, Research Diets, New Brunswick, NJ, USA) combining with intraperitoneal administration of diethylnitrosamine. The physical conditions and body weight of the animals were checked every week. If general fatigue, decreased activity, respiratory distress, or weight loss were observed, the mice were euthanized before the predetermined day. Through the above mentioned treatment, mice develop simple steatosis, NASH, and HCC in a definite manner under the condition that they were fed for the period of 36 weeks. We count on conventional histopathological findings such as steatosis, lobular inflammation, and ballooning as a common set of diagnostic criteria to confirm the actual types of pathology. During the experiments, mice may sometimes deteriorate their health states because of liver dysfunction. We prepared sections of mouse liver. These mice were sacrificed to obtain whole liver samples at 36 weeks after 12 h of fasting from the last feeding under general anesthesia by inhalation of 1–2% isoflurane. Small pieces of the liver were cut from the whole liver and then snap-frozen in liquid nitrogen immediately. After the sampling, animals were killed by cutting the inferior vena cava under deep anesthesia using isoflurane.

### Tissue slice preparation method

**Figure 1:**
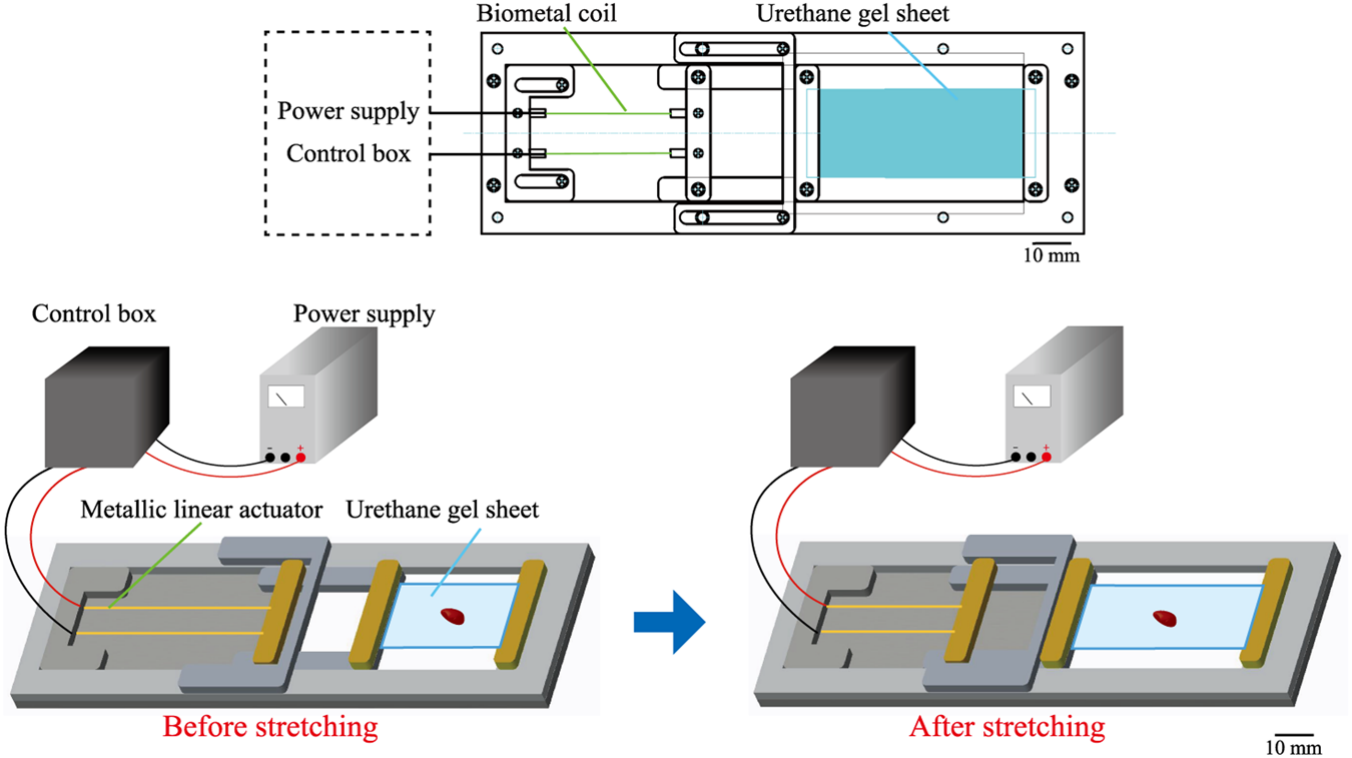
Schematic representation of the experimental set-up used to stretch a tissue slice attached to a gel sheet on the stage of an optical microscope.

To obtain a tissue sample of mouse liver, we applied the following treatment. First, we embedded a mouse liver in Optimal Cutting Temperature (OCT) compound immediately after the liver was removed from the mouse body and frozen in liquid nitrogen at −80 C°. The frozen sample was then sliced at a thickness of 20 µm using a Cryostat (Leica, Germany), which maintains samples at −20 C° for 15 minutes. Sample slices were then placed on a transparent urethane gel sheet (EXSEAL Corporation, Japan) in a careful manner so as to diminish any physical stress. It is known that polyurethane serves as tissue adhesive, and polyurethane-based sheets/scaffolds have been adopted to obtain tissue adhesive for application in tissue engineering^27^. Thus, in the present study we used urethane gel sheet as the adhesive material for the tissue slices. After treatment with Nile Blue (NB), the tissue slice was stood still for ca. 30 min at room temperature, and then we observed the tissue slice by optical microscopy. During such waiting period, the moisture contained in the sample slices is absorbed on the gel sheet, and as the result, the slices stick to the sheet in a strict manner. The tissue slices with the 10 × 10 mm size prepared were stretched with the experimental set-up as shown in Fig. 1. The urethane gel sheet measured 40 mm in length, 22 mm in width and 1 mm in thickness, and was fixed between a pair of bars, one fixed and one movable^28,29^. Extension was carried out through the use of metallic linear actuator (Toki Corporation, Japan), which shrink under the application of an electric current. In the experiments reported here, the gel sheet was stretched to 150%, from 40 mm to 60 mm. As for such degree of stretch, most of the stretch stress was forced on the urethane gel sheet. The tissue slices were monitored by optical microscopy using a 40x objective lens.

## Results

**Figure 2:**
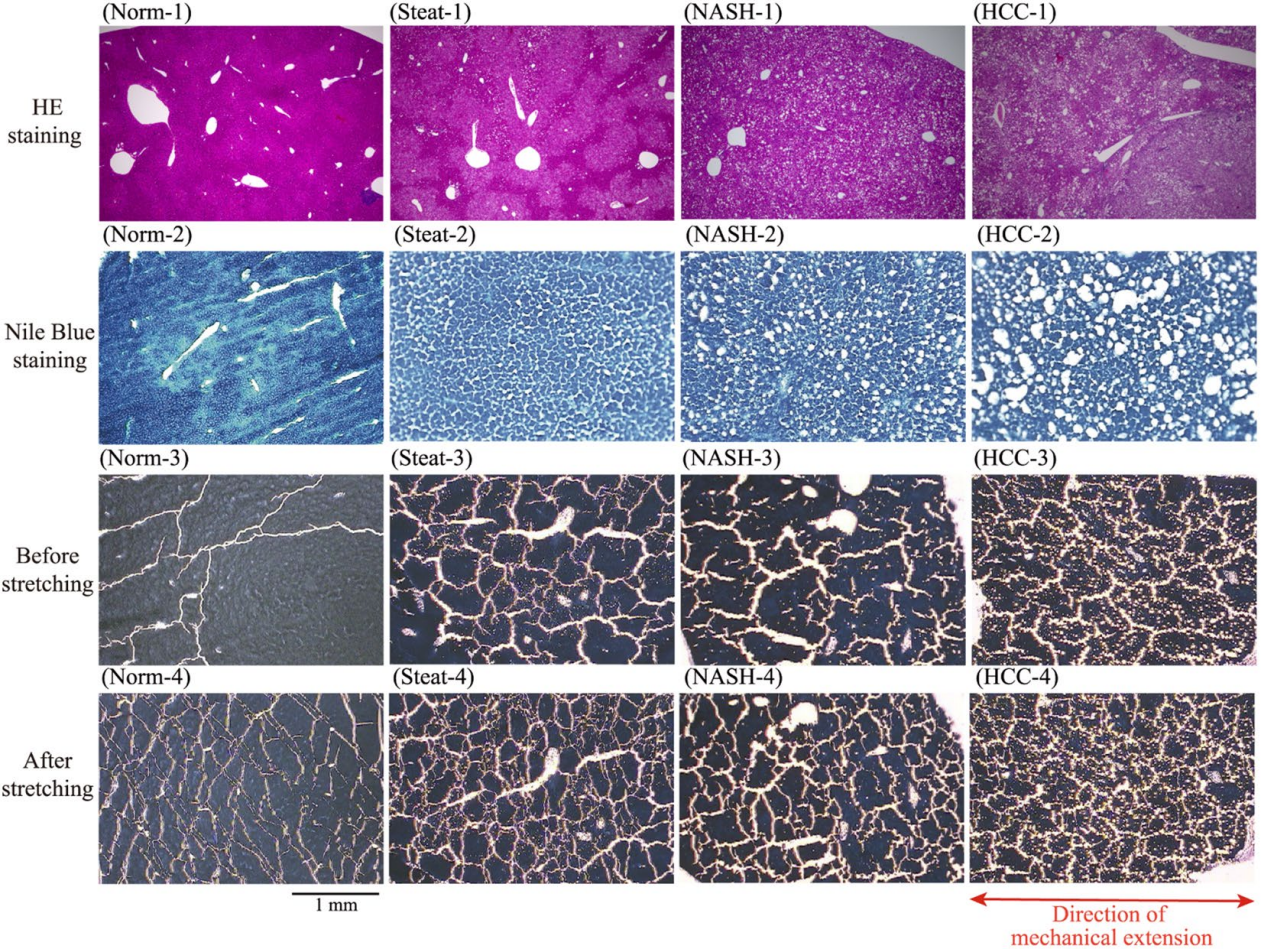
Optical microscopic images of tissue slices. (Norm-) healthy normal, (Steat-) simple steatosis, (NASH-) non-alcoholic steatohepatitis, (HCC-) hepatocellular carcinoma. From top to bottom: (1) HE staining on a glass slide, (2) NB staining on a glass slide, (3) Before stretching on a urethane gel sheet, (4) Stretched state with mechanical extension.

The upper rows in Fig. 2 show images of tissue samples of mouse liver on glass slides stained by Hematoxylin-Eosin (HE) (Norm-1, Steat-1, NASH-1, HCC-1) and Nile blue (NB) (Norm-2, Steat-2, NASH-2, HCC-2). These images indicate that there are rather significant differences between normal tissues and tissues with various stages of disease. However, it is not easy to clearly differentiate HCC (HCC-2) from simple steatosis (Steat-2) or NASH (NASH-2). The lower rows in Fig. 2 show images of tissue samples on a urethane gel sheet before (Norm-3, Steat-3, NASH-3, HCC-3) and after (Norm-4, Steat-4, NASH-4, HCC-4) stretching, under staining with NB. In this experimental set-up, the gel sheet to which tissue samples were attached was stretched to 150% (Norm-4, Steat-4, NASH-4, HCC-4). Comparison of the images obtained before and after stretching indicates a marked increase in tissue cracking under extension of the gel sheet. Here, it is noted that cracking appears even for the samples before the stretching. This is attributable to the dehydration effect caused through the hygroscopic nature of the gel sheet adhesive to the tissue slice. With the addition of the mechanical stress, cracking of the tissue is significantly enhanced as shown in Fig. 2(4). We have confirmed that the tissue slices kept to tightly stick to the gel sheet under stretch up to 150%. Interestingly, for the tissue with carcinoma (HCC-4), a fine cracking pattern is noted, whereas for the other tissues (Norm-4, Steat-4, NASH-4), the cracks appear as relatively straight lines without a fine pattern. Thus, stretching of the slices provides additional useful information specific to carcinoma; i.e., the appearance of a fine cracking pattern.

## Discussion

**Figure 3:**
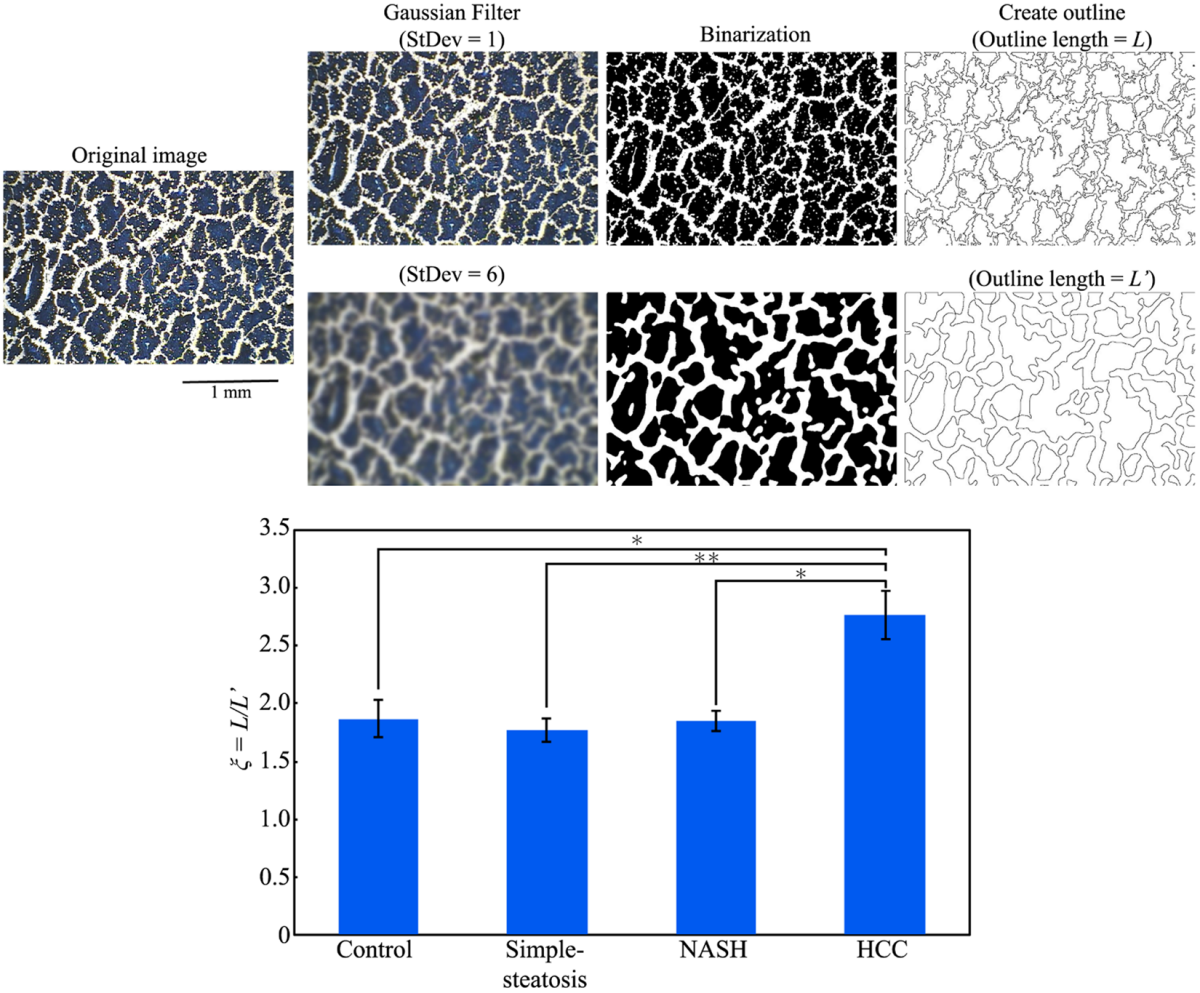
Schematic illustrations of the evaluation of uneven degrees of cracking lines. The total lengths of the outlines in binarized images before (*L*) and after (*L’*) Gaussian filtering for a length scale of 27.6 µm (corresponding to 6 pixels), where the distance between neighboring pixels in the original image is 4.6 µm. The bar graph shows the difference in *L/L’* for tissues with different disease states. (Mean ± SE, ^*^p < 0.05, ^**^p < 0.01, head count of the mouse *N* = 4).

The cracking patterns of the different tissues shown in Fig. 2 (Norm-4, Steat-4, NASH-4, HCC-4) varied according to the disease state, especially that of the cancerous tissue (HCC-4). Next, we will explore methods for the quantitative analysis of tissue cracking patterns toward future application for the diagnosis of disease states. We will adopt two different methodologies: i) evaluation of the degree of cracking in terms of the total length of cracks, and ii) evaluation of the degree of cracking in terms of the relative surface areas of cracks and uncracked tissues. We have applied Tukey-Kramer procedure to the statistical analysis on the experimental data, which is known as the standard procedure of analysis for multiple comparison^30–34^. Figure 3 shows the procedure used for image-analysis of the cracking pattern through the application of a Gaussian filter, with the different values of the standard deviation (StDev; i.e., 1 or 6 pixels) to smooth the lines in the cracking patterns, where the number of head count of the mouse *N* = 4. After the images were smoothed by Gaussian filtering, we binarized the images to sharpen the boundaries of the cracking patterns. The parts in white correspond to the cracking area of the samples after binarization. Next, we tried to evaluate the degree of the fineness in the cracking pattern based on the images with binarization where the distance between the neighboring pixels is 4.6 μm. By considering the characteristic size of individual cells on the order of 10 μm, we imposed Gaussian filter with the length scale of 27.6 μm (6 times larger than the distance between the neighboring pixels); in order words, the images were course grained above the size of individual cell. Then, we have evaluated the total lengths of the outlines of the cracking before and after the coursing and we have denoted these lengths as *L* and *L*’. The lower panel (Fig. 3) shows the ratio of the length *L* (StDev = 1 pixel) to *L’* (StDev = 6 pixels) for each disease state. The ratio *L/L’* for HCC is significantly greater (about 0.5-fold greater) than those for the other states. In other words, the roughening of the cracking pattern becomes significant in HCC; cancerous tissue. The micro-roughening on the cracking patterns as in Fig. 2 (HCC-3) and (HCC-4) are on the order of ten µm, corresponding to the usual cell-size in organs. In other words, the effect of coursing with the Gaussian filter is marked in HCC; cancerous tissue. As mentioned above, the length scale (27.6 μm) of the adapted Gaussian filter is larger than the cell-size in organs. Thus, the larger ratio of *L*/*L*’ implies the character of the cracking with the greater unevenness on the scale of individual cells. We may expect that the appearance of the fine unevenness on the cracking with the scale of the order of 10 μm is attributable to the decrease of cell-cell contact interaction^35,36^.

**Figure 4:**
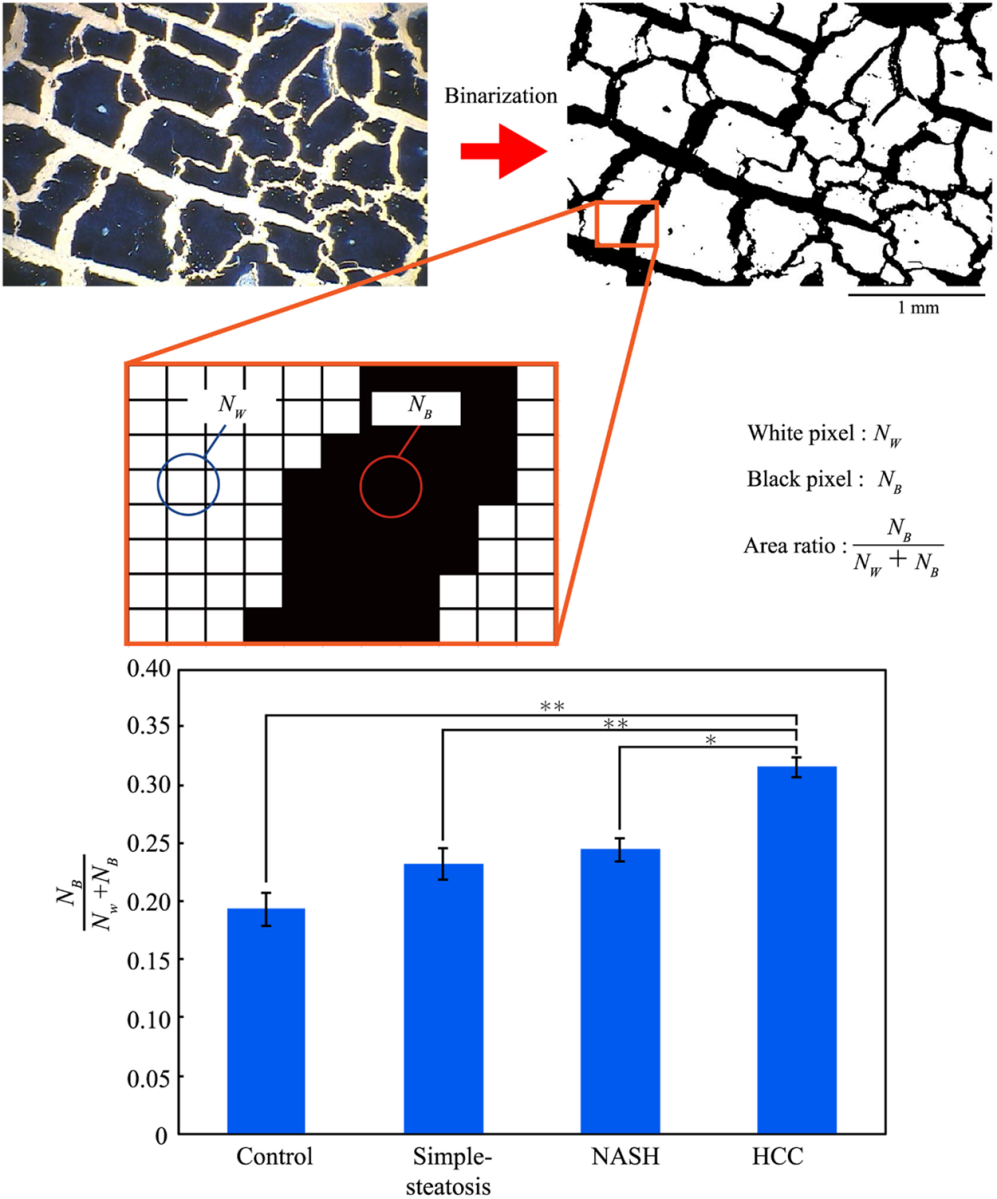
Schematic illustration of the evaluation of the cracking area. White and black pixels in the binarized image represent remaining tissue and cracks respectively. The bar graph shows the difference in the areas of cracks in tissues with different disease states. (Mean ± SE, ^*^p < 0.05, ^**^p < 0.01, head count of the mouse *N* = 4).

Figure 4 shows another method of analysis different from that in Fig. 3 that is based on an evaluation of the area in the cracking pattern, where the number of head count of the mouse *N* = 4. We applied this method to images that had been binarized. In the binarized image in Fig. 4, the black and white parts correspond to the urethane sheet without tissue and to remaining tissue respectively. To evaluate the relative area of cracking, we counted the numbers of white and black pixels after binarization; i.e., *N*_*w*_ for white pixels and *N*_*B*_ for black pixels, and then used these values to calculate the ratio of *N*_*B*_ to *N*_*w*_ + *N*_*B*_. This parameter increases gradually to the state of NASH. For tissue with HCC, this ratio is 0.5-fold greater than those for the other disease states. The increase of crack area in the stretched tissues is attributable to the weakening of adhesion strength between cells. These results show that it is possible to evaluate the state of a disease in a quantitative manner by using the parameters shown in Figs 3 and 4. The increase of the crack area in the stretched tissue of HCC is again attributable to the weakening of the cell-cell attractive interaction, as well as in the marked increase of *L*/*L*’ as in Fig. 3. Such weakening effect of the cell-cell adhesion on the other disease states beside HCC and, thus, cracking patterns did not reveal large differences among them.

## Conclusions

We have proposed a novel methodology for the pathological diagnosis of cancer, which involves making cracking patterns in tissue samples by stretching. We have shown experimental results together with the quantitative analysis on tissue samples of mouse liver in different disease states: Healthy normal state, Simple steatosis, Non-alcoholic steatohepatitis (NASH) and Hepatocellular carcinoma (HCC). It was revealed that the difference between NASH and HCC becomes significant for the tissues after mechanical stretching. The appearance of fine cracking on the order of cell-size in HCC implies the decrease of the strength of cell-cell attraction. Thus, the proposed methodology involving quantitative image-analysis on the cracking pattern is expected to serve as a useful diagnostic tool. In conclusion, cracking information with tissue stretching will be promising for the development of precise diagnosis, including malignancy in other tissues. Further studies on the cracking pattern formation by changing the experimental conditions, such as speed and magnitude of stretching, and method of freezing before tissue slicing, are awaited.
